# Two New Glycosides from the Fruits of *Morinda citrifolia* L

**DOI:** 10.3390/molecules171112651

**Published:** 2012-10-26

**Authors:** Ming-Xu Hu, Hong-Cai Zhang, Yu Wang, Shu-Min Liu, Li Liu

**Affiliations:** 1 Heilongjiang Academy of TCM, Heilongjiang University of Chinese Medicine, Harbin 150040, China; Email: humingxu204@yahoo.com.cn; 2 Academy of traditional Chinese medicine, Heilongjiang University of Chinese Medicine, Harbin 150040, China; Email: zhanghongcai-237@163.com (H.-C.Z.); 713358@sohu.com (Y.W.); 3 Department of Cardiology, the First Affiliated Hospital of Heilongjiang University of Chinese Medicine, Harbin 150040, China

**Keywords:** *Morinda citrifolia* L*.*, glycosides

## Abstract

To study the chemical constituents of the fruits of noni (*Morinda citrifolia* L*.*), and find novel compounds, an *n*-butanol extract of the ethanol soluble fraction was subjected to repeated silica gel and ODS column chromatography and HPLC. Two new glycosides were isolated and their structures elucidated by NMR and HRFAB-MS spectrometry as (2*E*,4*E*,7*Z*)-deca-2,4,7-trienoate-2-*O*-*β*-D-glucopyranosyl-*β*-D-glucopyra-noside (**1**) and amyl-1-*O*-*β*-D-apio-furanosyl-1,6-*O*-*β*-D-glucopyranoside (**2**), respectively.

## 1. Introduction

*Morinda citrifolia* L. (family Rubiaceae), also known as Tropical *Radix Morindae Officinalis* (RMO) and Medicinal Morinda Root Seasonal Fruit, is usually a small tree or bush occurring in the South Pacific tropical islands and widely distributed in the Hainan Province and Paracel Islands of China and in Taiwan. The fruits of *Morinda citrifolia* L. are oval and juicy with a strong odor, and have been used for a long time as a medicinal plant in Southeast Asia and the Pacific Islands. All parts of the plant can been used, including fruit, leaf, root, bark, flower, stem and seed [1]. Reported traditional uses include as a treatment of boils, abscesses, and inflammations of various origins, fungal infections, and constipation as well as diarrhea [2,3]. Pharmacological research has revealed a number of biological activities in recent years, such as anticancer [4], anti-inflammation [5], antioxidant [6], liver protection [7], and anti-AIDS properties [8]. In this work silica gel column chromatography was employed to separate the glucoside constituents of an *n*-butanol extract of the ethanol soluble fraction of *Morinda citrifolia* L. fruits. 2D-NMR techniques, HR-ESI-MS and hydrolytic reactions were used to elucidate the structures of the extracted compounds.

## 2. Results and Discussion

**Figure 1:**
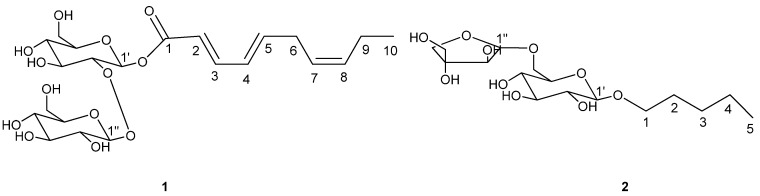
Structures of compounds **1** and **2**.

**Figure 2:**
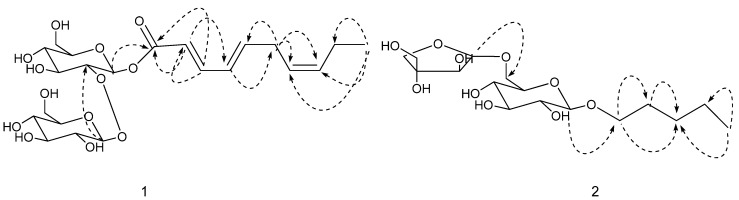
Key HMBC correlations of **1** and **2**.

Compound **1** (Figure 1) was obtained as a white powder (15.6 mg). The molecular formula was determined to be C_22_H_34_O_13_ by the HR-FAB-MS [M+H]^+^ peak at *m/z* 507.2071. Acid hydrolysis of **1** only gave D-glucose. The ^1^H and ^13^C-NMR spectra of **1** indicated an alkenoic acid ester moiety and two glucose groups. The alkenoic acid ester moiety was confirmed by ^1^H-NMR (Table 1) signals at δ_H_6.00 (1H, d, * J =* 15.2 Hz), 7.74 (1H, ddd, * J =* 15.2, 11.6, 0.8 Hz), 6.21 (1H, t, * J =* 11.3 Hz), 5.89 (1H, dt, *J =* 15.8, 7.8 Hz), 3.08 (2H, brt, * J =* 7.5 Hz), 5.33 (1H, m), 5.46 (1H, m), 2.12 (2H, dt, *J =* 7.5, 1.1 Hz), and 0.99 (3H, t, *J =* 7.5 Hz) and by ^13^C-NMR signals at δ_C_ 167.0 (s), 121.9 (d), 142.0 (d), 127.3 (d), 141.5 (d), 27.4 (t), 126.5 (d), 134.2 (d), 21.5 (t), and 14.6 (q). A combination of the COSY, HSQC, and HMBC data allowed assignment of the ^13^C-NMR (Table 1) signals from the disaccharide. The characteristic features of the two glucose moieties appeared in the ^13^C-NMR spectra, which exhibited signals at δ_C_ 94.5 (d), 83.0 (d), 77.6 (d), 71.2 (d), 77.8 (d), and 62.6 (t) for the first glucose and at δ_C_ 105.7 (d), 75.9 (d), 77.9 (d), 71.9 (d), 77.7 (d), and 62.2 (t) for the second glucose [9]. The ^1^H-NMR signals at δ_H_ 5.69 (1H, d, *J =* 7.8 Hz) and 4.53 (1H, d, *J =* 7.8 Hz), and the ^13^C-NMR signals at δ_C_ 105.7 (d) and 94.5 (d) indicated the presence of anomeric protons and carbons in the disaccharide moiety that had a β configuration according to the coupling constant. The HMBC correlation between the anomeric proton δ_H_ 4.53 (H-1′′) and δ_C_ 83.0 (C-2′) connected the terminal glucose to the inner glucose. The linkage between the fatty acid ester moiety and the disaccharide was also established by the HMBC correlation between anomeric proton δ_H_ 5.69 (H-1′) and δ_C_ 167.0 (C-1). Important HMBC interactions of compound **1** are shown in Figure 2. The coupling constant of 15.2 Hz between H-2 and H-3 indicated Δ^2,^^3^ to be *E* configuration. The coupling constant of 15.8 Hz between H-4 and H-5 indicated Δ^4,^^5^ to be *E* configuration. On the basis of the above data, the structure of **1** was deduced to be (2*E*,4*E*,7*Z*)-deca-2,4,7-trienoate-2-*O*-*β*-D-glucopyranosyl-*β*-D-glucopyranoside.

**Table 1:** NMR data of **1** and **2** in CD_3_OD (*δ* in ppm, *J* in Hz, recorded at 400 MHz and 100 MHz, respectively).

No.	1		2	
	δ_C_ (DEPT)	δ_H_ (*J*, Hz)	δ_C_ (DEPT)	δ_H_ (*J*, Hz)
1	167.0 (C)		71.4 (CH_2_)	3.56 m
2	121.9 (CH)	6.00 d (15.2)	32.4 (CH_2_)	1.53 m
3	142.0 (CH)	7.74 ddd (15.2,11.6,0.8)	24.5 (CH_2_)	1.24 m
4	127.3 (CH)	6.21 t (11.3)	23.4 (CH_2_)	1.24 m
5	141.5 (CH)	5.89 dt (15.8,7.8)	14.3 (CH_3_)	0.82 t (7.0)
6	27.4 (CH_2_)	3.08 br t (7.5)		
7	126.5 (CH)	5.33 m		
8	134.2 (CH)	5.46 m		
9	21.5 (CH_2_)	2.12 dt (7.5,1.1)		
10	14.6 (CH_3_)	0.99 t (7.5)		
1′	94.5 (CH)	5.69 d (7.8)	105.9 (CH)	4.45 d (7.8)
2′	83.0 (CH)		75.1 (CH)	
3′	77.6 (CH)		78.1 (CH)	
4′	71.2 (CH)		71.6 (CH)	
5′	77.8 (CH)		77.7 (CH)	
6′	62.6 (CH_2_)		68.4 (CH_2_)	
1′′	105.7 (CH)	4.53 d (7.8)	111.0 (CH)	4.86 d (2.3)
2′′	75.9 (CH)		78.0 (CH)	3.79 d (2.3)
3′′	77.9 (CH)		80.6 (C)	
4′′	71.9 (CH)		75.4 (CH_2_)	3.86 d (9.8) 3.64 d (9.8)
5′′	77.7 (CH_2_)		65.7 (CH_2_)	3.47 s
6′′	62.2 (CH_2_)			

Compound **2** (Figure 1) was obtained as a white powder (17.9 mg). The molecular formula was deduced from the HR-FAB-MS 383.1911 [M+H]^+^ and the ^13^C-NMR data to be C_16_H_30_O_10_. Acid hydrolysis of **2** only gave D-glucose and apiose. The ^1^H- and ^13^C-NMR spectra of **2** indicated a heptanol moiety, a glucose group and an apiose group. The heptanol moiety was supported by ^1^H-NMR signals at δ_H_ 3.56 (2H, m), 1.53 (2H, m), 1.24 (4H, m), and 0.82 (3H, t, *J* = 7.0 Hz) and by ^13^C-NMR signals at δ_C_ 71.4 (t), 32.4 (t), 24.5 (t), 23.4 (t), and 14.3 (q). A combination of the COSY, HSQC, and HMBC data allowed assignment of the ^13^C-NMR signals of the disaccharide. The characteristic features of the glucose moiety and the apiose moiety appeared in the ^13^C-NMR spectra, which exhibited signals at δ_C_ 105.9 (d), 75.1 (d), 78.1 (d), 71.6 (d), 77.7 (d), and 68.4 (t) for the glucose and signals at δ_C_ 111.0 (d), 78.0 (d), 80.6 (s),75.4 (t), 65.7 (t) for the apiose [10]. The HSQC correlation between anomeric proton δ_H_ 4.45 (1H, d, *J* = 7.8 Hz) and anomeric carbon δ_C_ 105.9 (d) indicated that the glucose moiety had a β configuration. The HSQC correlation between anomeric proton δ_H_ 4.86 (1H, d, *J* = 2.3 Hz) and anomeric carbon δ_C_ 111.0 (d) indicated that the apiose moiety had a β configuration. The HMBC correlation between anomeric proton δ_H_ 4.86 (H-1′′) and δ_C_ 68.4 (C-6′) connected the terminal glucose to the inner glucose linkage. The linkage between the fatty acid ester moiety and the disaccharide was also established by the HMBC correlation between anomeric proton δ_H_ 4.45 (H-1′) and δ_C_ 71.4 (C-1). Important HMBC interactions of compound **2** are shown in Figure 2. On the basis of the above data, the structure of **2** was deduced to be amyl-1-*O*-*β*-D-apiofuranosyl-1,6-*O*-*β*-D-glucopyranoside.

## 3. Experimental

### 3.1. General

IR and NMR spectra were recorded on Shimadzu FTIR-8400S and Bruker DPX 400 (400 MHz for ^1^H-NMR and 100 MHz for ^13^C-NMR) instruments, respectively. Chemical shifts are given as δ values with reference to tetramethylsilane (TMS) as an internal standard, and coupling constants are given in Hz. The HR-ESI-MS analyses were conducted on a Waters LCT Premier XE TOF-MS instrument. A Hypersil ODS II (5 μm, 4.6 × 250 mm, Dikma, Lake Forest, CA, USA) column was employed for analytical HPLC (Waters, 2695-2998 instrument). Preparative HPLC (Agilent 1100 system, Santa Clara, CA, USA) was performed on a Pegasil ODS II (5 μm, 9.4 × 250 mm, Agilent) column. Silica gel (200–300 mesh, Haiyang, Qingdao, China) was employed for column chromatography and ODS-A (120 A, 50 μm) was obtained from YMC Co. (Kyoto, Japan).

### 3.2. Plant

The fresh fruits of *Morinda citrifolia* L. were collected from Hainan Province of China, in July 2010. The voucher specimen (20100720) was deposited in Heilongjiang University of Chinese Medicine, Harbin, China.

### 3.3. Extraction and Isolation

The fruits of *Morinda citrifolia* L. (26 kg) were ground and sieved through standard mesh sieve No. 10 and extracted with 95% EtOH (3 × 10 L) for 2 h. Concentration under reduced pressure gave the EtOH extract (210 g) which was dissolved in water (10 L), and successively extracted with petroleum ether (60–90 °C), EtOAc, and *n*-butanol, (3 × 10 L) respectively. Solvents were removed to give the petroleum ether (20.2 g), CHCl_3_ (5.7 g), EtOAc (6.9 g) and *n*-butanol (162.4 g) extracts. The *n*-butanol fraction was repeatedly column chromatographed on silica gel with a gradient of CHCl_3_/MeOH (1:0→0:1) as eluent to afford five fractions: Fr_1_ (6 g), Fr_2_ (13 g), Fr_3_ (16 g), Fr_4_ (8 g), Fr_5_ (21 g), Fr_6_ (10 g), Fr_7_ (4 g), and Fr_8_ (24 g). 

Fr_3_ (2.6 g) was subjected to ODS column chromatography with MeOH/H_2_O (1:9→1:0) and finally purified by preparative HPLC on a Pegasil-ODS Ⅱ column with MeOH/H_2_O (2:8) to afford **1** (15.6 mg, t_R_ = 43 min). Fr_5_ (21 g) was subjected to repeated silica gel chromatography with CHCl_3_/MeOH (30:1→0:1) elution to afford a number of subfractions B_1_-B_4_. B_2_ (2.7 g) was subjected to ODS column chromatography with MeOH/H_2_O (1:9→1:0) and finally purified by preparative HPLC on a Pegasil-ODS Ⅱ column with MeOH/H_2_O (3:7) to afford **2** (17.9 mg, t_R_ = 28 min). 

*(2E,4E,7Z)-deca-2,4,7-trienoate-2-O-β-D-glucopyranosyl-β-D-glucopyranoside* (**1**). White amorphous powder. mp. 132–134 °C. IR (KBr): 3396.3, 1740.6, 1472.1, 1071.6, 988.4, 905.3 cm^−1^. HR-ESI-MS *m/z* 507.2071 [M+H]^+^ (calc. C_22_H_34_O_13_, 507.2078); ^1^H and ^13^C-NMR (CD_3_OD) data see Table 1.

*Amyl-1-O-β-D-apiofuranosyl-1,6-O-β-D-glucopyranoside* (**2**). White amorphous powder. mp. 77–79 °C. IR (KBr): 3403.1, 1469.3, 1375.8, 1071.4 cm^−1^. HR-ESI-MS *m/z* 383.1911 [M+H]^+^(calc. C_16_H_30_O_10_, 383.1917); ^1^H and ^13^C-NMR (CD_3_OD) data see Table 1.

## 4. Conclusions

Two novel glucosides were isolated from the *n*-butanol fraction of *Morinda citrifolia* L. fruits. Compound **1** is (2E,4E,7Z)-deca-2,4,7-trienoate-2-O-*β*-D-glucopyranosyl-*β*-D-glucopyranoside and compound **2** is amyl-1-O-*β*-D-apiofuranosyl-1, 6-O-*β*-D-glucopyranoside.
